# The NLRP3 Inflammasome Inhibitor Dapansutrile Attenuates Cyclophosphamide-Induced Interstitial Cystitis

**DOI:** 10.3389/fimmu.2022.903834

**Published:** 2022-06-03

**Authors:** Sonia Kiran, Ahmed Rakib, Udai P. Singh

**Affiliations:** Department of Pharmaceutical Sciences, College of Pharmacy, The University of Tennessee Health Science Center (UTHSC), Memphis, TN, United States

**Keywords:** Cystitis, mast cells, bladder inflammation, NLRP3 inflammasome, urinary bladder, macrophages

## Abstract

Interstitial cystitis (IC)/bladder pain syndrome (BPS), hereafter referred together as IC, is a clinical syndrome characterized by sterile inflammation in the bladder. While the etiology and pathophysiology of IC remain unclear, it may involve autoimmunity in light of the significant role played by the NLRP3 inflammasome. However, the effect of NLRP3 inhibitors including dapansutrile (Dap) on IC had not been explored previously. Here, we investigated the effect of Dap in the cyclophosphamide (CYP)-induced experimental mouse model of IC, which results in functional and histological alterations confined to the urinary bladder (UB) comparable to that of clinical IC. CYP-induced mice treated with Dap exhibited improved UB pathology and reductions in inflammation scores and the frequency and the number of mast cells and neutrophils, relative to mice that received CYP alone. Dap- and CYP-treated mice also exhibited infiltration of T cells in the spleen and iliac lymph nodes (ILNs) and a concurrent significant decrease (p<0.01) in CXCR3^+^CD8^+^ T cells in the UB, induction of systemic and mucosal dendritic cells (DCs), and reduced levels of systemic proinflammatory cytokines, as compared to CYP alone. We also observed decreases in the expression of several signaling pathways regulators, including interleukin-1 beta (IL-1β), NLRP3, caspase-1, nuclear factor kappa B (NF-κB), and inducible nitric oxide synthase (iNOS) in the UB of CYP- and Dap-treated mice, relative to those receiving CYP alone. Taken together, these results suggest that Dap suppresses IC through the reduction of CXCR3^+^T cells, mast cells, and neutrophils in the UB and induces DCs as a protective measure. The present study identifies the mechanisms underlying the amelioration of IC by the NLRP3 inhibitor Dap and may provide an avenue for a potential therapeutic agent for the treatment of IC.

## Introduction

Interstitial cystitis (IC)/bladder pain syndrome (BPS), hereafter referred together as IC, is characterized by symptoms of the lower urinary tract (LUT), including chronic pain, bladder discomfort, and pelvic tenderness. The number of IC cases continues to increase and currently numbers ~6,530 females and ~4,200 males per 100,000 persons diagnosed with IC (1). While the precise etiology of IC remains unclear, increasing evidence supports the roles of chronic stress, infection, and immune modulation in exacerbating IC. Consequently, most IC patients reporting inconsistent pain and urinary void frequency were further negatively impacted due to stressful conditions (2, 3). Urinary bladder (UB) urothelial dysfunction and inflammation in the urothelium are also associated with IC (4), suggesting a role for immune modulation in the UB. Epithelial dysfunction causes abnormal infiltration of mast cells, neutrophils, dendritic cells (DCs), and activated T cells, all of which interact with components of the urothelium to cause UB inflammation (1). Moreover, infection by *Escherichia coli* releases lipopolysaccharide, which leads to further activation of inflammasomes and releases pro-inflammatory cytokines and interleukin (IL)-1β, which also enhances inflammation (5, 6).

The principal etiology of IC involves this increase in the numbers of T cells and mast cells and the local adaptive immune response in the UB (7). Urothelial antigen-specific CD4 T cells serve as direct effector cells to induce bladder autoimmune inflammation (8). Experimental and clinical IC are both characterized by an increase in the number of mast cells below the epithelial layer (9), where they produce a variety of cytokines and chemokines, including the CXCR3 ligand CXCL9, which attract neutrophils (10), T cells (11), and partially contribute to bladder injury (12). Mast cells also secrete several cytokines, leading to visceral and neurogenic inflammation (13). Submucosal macrophages increase in number during IC and induce inflammation by enhancing the production of inducible nitric oxide synthase (iNOS) by inflammatory cells, thereby altering the histopathology of the urothelium (14). Interestingly, in a model of arthritis, the number of T cells that express CXCR3 increases, while blocking the interactions of CXCR3 with other molecules suppresses the severity of arthritis (15). Similarly, the results of our previous studies in CYP-induced cystitis suggest that activated T cells infiltrate the bladders of mice (16). Therefore, UB inflammation emerges as a key element in the pathology of IC. Thus, determining the precise tool to suppress UB inflammation and autoimmunity during IC will lead to better treatment options.

To date, no single effective therapeutic approach has been developed for IC, despite the application of several unsuccessful approaches. Recently Chuang et al. (2020) utilized extracorporeal shock wave therapy (ESWT) as a remedy for IC but no thorough investigation has been performed to optimize the dosage and effects of ESWT treatment on IC (17). Therapeutic agents such as amitriptyline, pentosan polysulfate (PPS), and cyclosporin have been used with some partial success, but the majority of these drugs have adverse side effects (18). For example, treatment of IC with amitriptyline leads to weight gain, feelings of sedation, and drowsiness (19).

The thioredoxin antioxidant system is negatively regulated by thioredoxin interacting protein (TXNIP) (20). TXNIP is vital in the oxidative stress processes of many diseases, including acute lung injury (21), atherosclerosis (22), and ischemia-reperfusion injury (23). In IC, inhibition of TXNIP activity may reverse oxidative stress (24) *via* a pathway that involves the NOD-, LRR- and pyrin domain-containing protein 3 (NLRP3) inflammasome (1). The NLRP3 inflammasome can be activated by several different substances, including molecules related to injury, bacterial infection, and other environmental stimuli (25, 26). TXNIP links NLRP3 inflammasomes with linking reactive oxygen species (ROS), thereby serving as a connecting bridge between inflammation and oxidative stress. Inflammation is enhanced by activation of the proinflammatory transcription factor nuclear factor-κB (NF-κB), which mediates the release of pro-inflammatory factors including tumor necrosis factor-alpha (TNF-α) (27), activates NLRP3 transcription, and stimulates inflammasome formation (28). NLRP3 inflammasomes also recruit and activate the protease caspase-1, leading to the release of the proinflammatory cytokine interleukin 1 beta (IL-1β) (29). Thus TXNIP, NLRP3, and NF-κB may provide druggable targets for the development of novel treatments for IC.

In the cyclophosphamide (CYP)-induced mouse model for IC, intraperitoneal (i.p.) injection of CYP increases levels of NLRP3 inflammasomes that mediated bladder injury and inhibition of NLRP3 could effectively reverse this damage (30). For example, the NLRP3 inhibitor dapansutrile (Dap) ameliorates inflammation-related immune cell infiltration in experimental autoimmune encephalomyelitis (30) and lowers the metabolic cost of inflammation (31). However, the mechanism by which NLRP3 inhibitors such as Dap suppress the severity of IC symptoms and alter the immune response in the UB remains elusive until now. Therefore, the goal of this study is to determine how Dap attenuates CYP-induced IC in mice.

We report here that Dap effectively eases the severity of IC by inducing dendritic cell (DC) numbers in systemic and mucosal organs, reducing numbers of inflammatory mast cells, activated T cells, and neutrophils, and decreasing levels of systemic cytokines *via* the caspase-1 and NF-κB pathways, thereby decreasing levels of iNOS and IL-1. Our study supports the efficacy of Dap as a potential treatment for IC and provides an opportunity for developing novel therapeutic agents for clinical IC.

## Materials and Methods

### Mice

Female C57BL/6 mice strain at age ~8 weeks and weighing 18-20 grams were procured from Jackson Laboratories (Bar Harbor, ME). All mice were maintained in specific cages under normal 12:12 h light and dark cycles for a week of acclimatization in conventional housing conditions at the University of Tennessee Health Science Center (UTHSC) laboratory animal care unit. All possible efforts were made to reduce animal pain and stress. The UTHSC Institutional Animal Care and Use Committee (IACUC) approved all animal experimental protocols used in this study (UPS# 20-0169). Power analysis indicated that six mice per group would allow detection of effect sizes and standard deviations between groups at a significance level of 5% and power of 80% using two-sided t-tests or within the context of ANOVA. At the end of the week of acclimatization, the mice were randomly divided into three groups (n=6 mice/group): Control, CYP+vehicle, or CYP+Dap). Each study was repeated three times to ensure statistical relevance.

### Cyclophosphamide Induced IC

The cytostatic agent cyclophosphamide (CYP; ICN Biomedicals, Aurora, OH) is commonly used to cure bladder-toxic neoplastic diseases and stimulate hemorrhagic IC. To induce experimental IC in mice, animals were injected with CYP (100 μl containing 80mg/Kg body weight) *via* the i.p. route on alternate days (1, 3, 5, and 7), while the control group received 100 μl of saline on the same schedule, until the experimental endpoint on day eight. We monitored the mice daily for changes in body weight and behavior (grooming, guarding) that could reflect the symptoms of IC.

### Pharmacological Treatments of Dapansutrile 

The NLRP3 inflammasome inhibitor dapansutrile (Dap; Tocris R&D systems, Minneapolis, MN) was administered to the CYP+Dap group of mice (100μl containing 100mg/kg body weight) *via* oral gavage every other day (2, 4, 6, and 8) during the CYP treatment until the experimental endpoint on day eight. As noted above, we monitored the mice daily for changes in body weight and behavior (grooming, guarding) that could reflect the symptoms of IC.

### Single-Cell Suspensions From Spleen, Iliac Lymph Nodes, and UBs 

At the experimental endpoint, whole blood was collected (see below) and all mice were euthanized on day eight following isoflurane inhalation. The spleen, iliac lymph nodes (ILNs), and UB of each mouse were harvested individually and each was dissociated using a stomacher (Seward Stomacher^®^ 80) to make a single-cell suspension. Red blood cells (RBCs) were eliminated from the spleen with the help of lysis buffer (Thermo Fisher Scientific). Mouse UB cells were separated using a multi-tissue dissociation kit. In brief, the UB was collected and gently placed in MACS C tubes (MACS Miltenyi Biotec, 130-096-334) and processed using a multiple tissue dissociation kit (MACS Miltenyi Biotec, 130-110-201) with the help of a gentle MACS™ Dissociator (MACS Miltenyi Biotec, 130-093-235). Cell debris was removed using a cell strainer with 70μ mesh (Sigma, St. Louis, MO), and UB cells were washed with RPMI 1640 medium containing 10% fetal bovine serum (FBS), and placed at 4°C for use in flow cytometry later on the same day, within two hours of isolation.

### Flow Cytometry Staining and Analysis

Fluorescent-conjugated antibodies, their respective isotype controls, and compensation beads were obtained from Bio-Legend (San Diego, CA). Cells were isolated from the spleens, ILNs, and the UBs from each mouse. Cells were stained by resuspending them in 80µl of ice-cold flow cytometry staining buffer (PBS containing 1% FBS) and 5µl of each fluorescent-conjugated antibody or their respective controls for 40 min at 4°C. All antibodies were mouse monoclonals: anti-CD 11b (clone M1/70), anti-CD 11c (clone N418), anti-CD3 (clone 12A2), anti-Ly6c (HK 1.4), anti-CD8 (clone 5367), anti-CD4 (clone GK 1.5), CXCR3 (clone CXCR3-173), CD117/c-kit (clone 2B8), and FCϵR1α (clone MAR-1). After incubation, the cells were thoroughly rinsed and resuspended in 300µl of FACS buffer and analyzed using an Agilent Novocyte flow cytometer.

### Changes in Cytokine and Chemokine Levels During IC

Whole blood samples were taken at the time of sacrifice as noted above, sera were collected by centrifugation and stored at -80°C before analysis. Systemic levels of proinflammatory and anti-inflammatory cytokines and chemokines were determined simultaneously using the Milliplex^®^ MAP mouse cytokine/chemokine magnetic beads kit (MCYTOMAG-70K; Millipore Sigma). The 96-well detection plates were washed with wash buffer before 25µl of standards, controls, or serum samples from control, CYP+vehicle, and CYP+Dap groups of mice were added to the proper wells and 25µl of beads were added to each well. The plate was incubated at 4^0^C on continuous shaking, the solute was gently removed from each well by aspiration, and the plate was washed twice with a magnetic plate washer. A detection antibody (25µl) was added, the plate was incubated at room temperature (RT) for 1 h with continuous shaking, and streptavidin-phycoerythrin (25µl) was also added to some wells. The plate was wrapped with foil and incubated at RT on a shaker for 30 min. Finally, the contents of each well were removed by aspiration, the wells were washed using a plate washer, sheath or drive fluid (150µL) was added, and the plate was read using an LX200 Luminex instrument. The data was analyzed and the cytokine/chemokine concentrations were calculated and graphed with GraphPad Prism software.

### Immunoblot Analysis

The protein in pooled UB cell lysates from each group was analyzed by immunoblot. Protein concentrations in each lysate were determined using a BCA Protein Assay Kit (Pierce; ThermoFisher Scientific) and 20µg of protein from each group was separated on a precast 12% sodium dodecyl sulfate (SDS) gel until the dye front reached the bottom of the gel. Proteins were transferred from the SDS-PAGE gel to a PVDF membrane (#1620174, Bio-Rad) using a Trans blot turbo instrument (Bio-Rad). Membranes were blocked with intercept blocking buffer (#92760001, LI-COR Biosciences USA) at RT for 1 h and incubated with primary antibodies overnight at 4°C with shaking. Primary antibodies used were anti-IL-1β (1:1000, 3A6, Cell Signaling), anti-Caspase 1 (1:1000, E2Z1C, Cell Signaling), anti-NLRP3/NALP3 (1:200, # AG-20B-0014-Cryo-2, AdipoGen Life Sciences, San Diego, CA), anti-NOS2 (1:200, #SC-7271, Santa Cruz Biotechnology), anti-NF-κB p50 (1:200, #8414, Santa Cruz Biotechnology), and anti-β-actin (1:1000) (#926-42212, LI-COR Biosciences). Membranes were washed (three times 5 min each) in PBS containing 0.2% Tween 20 and incubated with IRDye^®^ 800CW-labeled goat anti-mouse IgG (#926-32210, LI-COR Biosciences), IRDye^®^ 680RD-labeled goat anti-mouse IgG (#925-68070, LI-COR Biosciences), or goat anti-rabbit IgG (#926-32211, LI-COR Biosciences) secondary antibodies (1:5,000) at RT for 1 h. Images were scanned using the LI-COR Biosciences Odyssey Sa Infrared Imaging system and densitometry analyses were performed with LI-COR Image Studio Software.

### Histology

The bladder sections of individual mice from three independent groups (control, CYP+vehicle, and CYP+Dap) were rinsed with PBS, fixed in fresh 10% paraformaldehyde, and embedded in paraffin. The tissues were sectioned at 4μm and stained with hematoxylin and eosin (H&E) in UTHSC’s histology core facility. The sections were examined microscopically at magnifications of 40X and scored for the severity of cystitis. The data presented are the mean ± SEM percentage of changes in each experimental group. The inflammatory state of the three groups (control, CYP+vehicle, and CYP+Dap) UBs was characterized and scored as described earlier (32). In brief, the scoring was as follows: 0-1, no to a few (<10) mononuclear cellular infiltrates and no signs of inflammation in the UB; 2-3, minimal hyperplasia with a mixture of (<50) mononuclear cells and various immune cellular infiltrates; 4-5, major hyperplasia and a large number of cellular infiltrates in the UB lamina propria; 5-6, major hyperplasia, epithelial cell erosion, a large number of cellular infiltrates, and inflammation in the sub-mucosa.

### Statistical Analysis

Several response variables were measured for each experimental unit within the three groups (control, CYP+vehicle, and CYP+Dap) and the mean values and standard error of the sample mean (SEM) were calculated. T-tests within the context of ANOVA were used to show whether any differences in mean cytokine/chemokines levels between the three groups were statistically significant. The results were analyzed using the Statview II statistical program (Abacus Concepts, Inc., Berkeley, CA) for Macintosh computers and were considered statistically significant if p values were less than 0.05 (^*^ *p<0.05;* ^**^ *p*<0.01; ^***^ *p*<0.001).

## Results

### Changes in Body Weight, Wet Bladder Weight, Inflammation Score, and Histopathology During CYP-Induced IC

**Figure 1:**
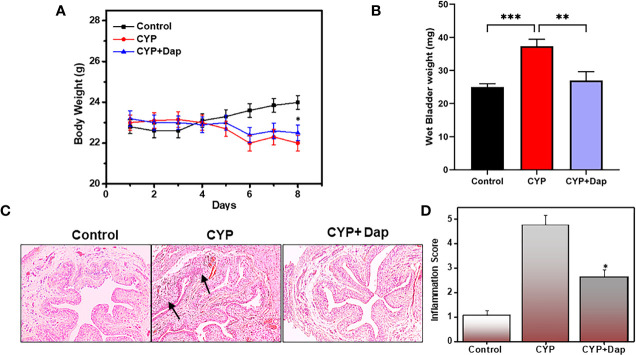
Changes in body weight, bladder weight, inflammation score, and urinary bladder (UB) histology after Dap treatment during CYP-induced IC. C57BL/6 control mice (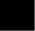) received normal water, (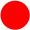) received CYP+vehicle, and (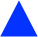) received CYP+Dap (100 mg/kg body weight of dapansutrile every alternate day beginning at day two, while both CYP+vehicle and CYP+Dap groups received CYP continually every alternate day beginning at day one until day eight. The body weight of the mice was recorded every day, and any body weight changes are expressed in **(A)** Mice were sacrificed after eight days and the wet bladder weights of the three groups of mice are presented in **(B)** Histological sections of UBs from the three groups of mice are shown in **(C)** Mice that received CYP+vehicle showed cellular infiltration and reduced lumen space as compared to control mice **(C**; arrow**)**, while mice that received CYP+Dap showed significantly reduced cellular infiltrations and lower inflammation scores **(D)**. All data represent the mean value ± SEM from three independent experiments involving six mice per group (n=18). Asterisks indicate statistically significant differences between CYP+vehicle- and CYP+Dap-treated groups (^*^p<0.05 and ^**^p<0.01) or statistically significant differences between control and CYP induced wet bladder weight (^***^p<0.001).

We examined changes in body weight and wet bladder weight of mice in the control, CYP+vehicle, and CYP+Dap treated groups. While we did not observe any statistically significant changes in body weight between vehicle-treated mice and mice treated with Dap after CYP induction, CYP treatment reduced body and wet bladder weight relative to the control group (P<0.05). Dap treatment slightly improved body weight (**Figure 1A**) and improved wet bladder weight (**Figure 1B**), relative to CYP alone. Changes in urinary bladder (UB) pathology and inflammation score were examined in all three groups of mice at the experimental endpoint. The UB of control mice exhibited normal UB pathology with only limited (<10) inflammatory immune cell infiltrates (**Figure 1C**), while the mice that received CYP+vehicle exhibited extensive small to multifocal cellular infiltrates comprised primarily of T cells, mast cells, and neutrophils (**Figure 1C** middle panel) relative to the control. In mice treated with both CYP and Dap, the cellular infiltrates and IC inflammatory score was reduced compared to the CYP+vehicle group (**Figure 1D**). Taken together, these results suggest DAP treatment reduced the severity and inflammatory pathology of CYP-induced IC.

### Dap Modulates Systemic and Mucosal T Cell Responses

**Figure 2:**
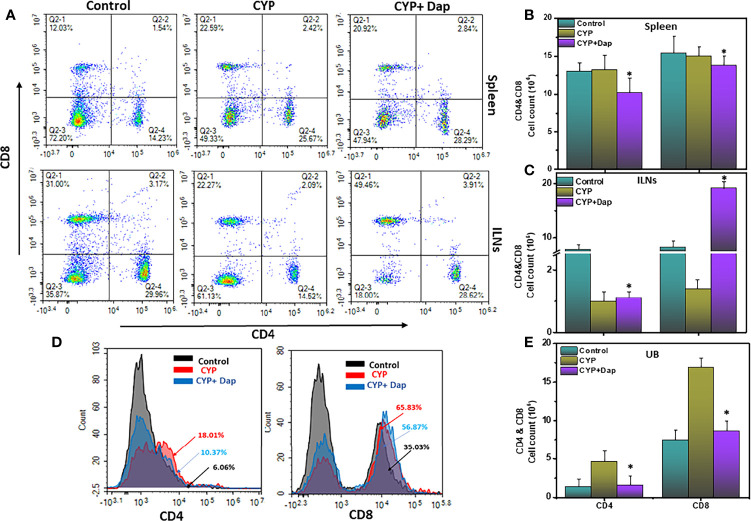
Dap mediates the frequency of systemic and mucosal T cells. Single-cell suspensions from spleen, iliac lymph node (ILN), or UB were isolated from each group on day eight, stained for T cell phenotypic markers, and analyzed by flow cytometry. Panels A shows a representative experiment indicating the percentages of CD4^+^T cells **(A**, lower right quadrant**)** and CD8^+^T cells **(A**, upper left quadrant**)**. **(D)** shows the percentage of both CD4^+^ T cells and CD8^+^T cells as histogram plots in UBs. **(B, C, E)** show numbers of CD4^+^T and CD8^+^T cells in the spleen, ILNs, and UBs respectively. Data shown in **(B, C, E)** represent the total number of these cells ± SEM from three independent experiments involving six mice per group (n=18). Asterisks indicate statistically significant differences between CYP+vehicle and CYP+Dap treated groups (^*^p < 0.05).

We used flow cytometry to determine the effect of Dap treatment on the populations of T cells in the spleens, ILNs, and UBs of mice with CYP-induced IC. After CYP induction, the frequency of CD4 and CD8T cells increased in the spleens and UBs and decreased in the ILNs relative to controls. Dap treatment had the opposite effect in increasing the frequency and numbers of CD4 and CD8T cells in ILNs (**Figures 2A–C**) and reducing them in the UBs and numbers in the spleen (**Figures 2D, E**). These findings suggest that in CYP-induced IC, there is a considerable reduction of T cells in ILNs but an increased frequency of T cells in the UBs. However, Dap treatment reduces the frequency and number of both T cells in the UBs, which might be in part responsible for reducing the severity of IC.

### Dap Treatment Reduces the Frequency of the CD8^+^CXCR3^+^T Cell in the UB

**Figure 3:**
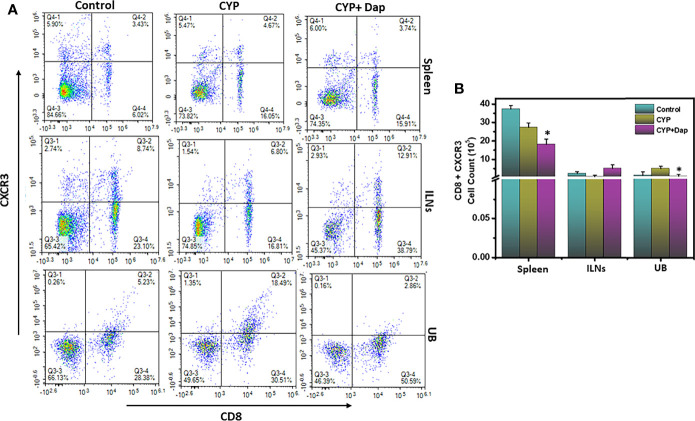
Dap treatment reduces the systemic and UB CXCR3+ T cells. Single-cell suspensions from spleen, ILN, and UB were isolated from the three groups as in the legend in **Figure 2** and stained for T cell activation markers using anti-CXCR3 Abs. **(A)** shows a representative experiment indicating the percentages of CD8+CXCR3+ cells (A, upper right quadrant). **(B)** shows Mean values ± SEM from three independent experiments involving six mice per group (n=18) are shown.Asterisks indicate statistically significant differences between CYP+vehicle and CYP+Dap treatment groups (*p < 0.05).

The chemokine receptor CXCR3 facilitates T cell differentiation, induces the generation of both effector and memory T cells, and is important for the proper functioning of T cells and mast cells. The increased expression of CXCR3 on the surface of T cells is also an indicator of their activation in various inflammatory conditions disease models. In this study, we observed a significant reduction (p<0.01) in the numbers and frequency of CXCR3^+^expressing CD8 T cells in the Dap treated group spleen and UBs as compared to CYP alone (**Figures 3A, B**). This reduction in activated CD8^+^CXCR3^+^ T cells in the UBs after Dap treatment might account for reductions in the severity and inflammation during IC.

### Dap Treatment Affects the Frequency and Distribution of Dendritic Cells

**Figure 4:**
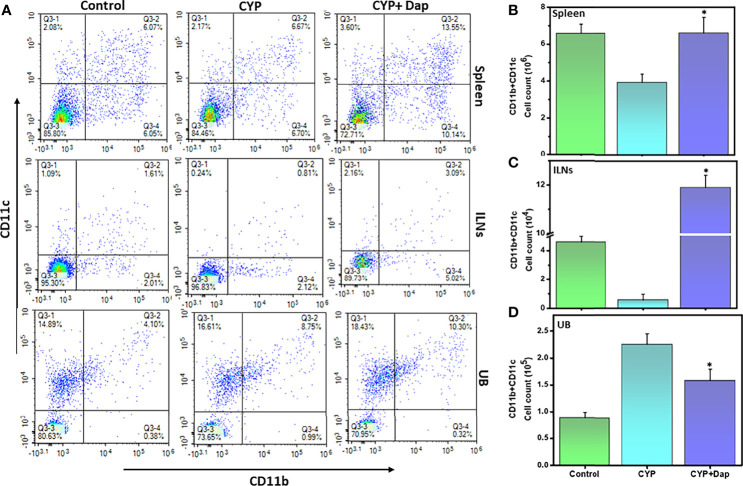
Dap alters dendritic cells (DCs) frequency during IC. Single-cell suspensions from the spleen, ILN, and UB were isolated from the three groups at the experimental endpoint, as described in the legend in **Figure 2**. Mean values ± SEM from three independent experiments involving six mice per group (n=18) are shown. Changes in the frequency and expression of DCs (CD11b^+^CD11c^+^) from spleens, ILNs, and UBs cells are shown in **(A)**, while changes in the number of DCs in the spleen, ILN, and UBs are shown in **(B–D)**, respectively. The statistical significance of flow cytometry data between the three groups was assessed and asterisks indicate statistically significant differences between CYP+vehicle and CYP+Dap groups (^*^p<0.01). Representative data from one of at least three experiments that produced similar results are depicted in the figure.

DCs can engulf antigens, process them, and present them to T cells. During inflammation, DCs migrate from non-lymphoid to lymphoid tissues for activation of naïve T cells. DC activation is a critical step during inflammation. To better define the role of Dap in mediating DCs during IC, we assessed changes in frequency and number of DCs (CD11b^+^ CD11c^+^) in spleens, ILNs, and UBs of mice after CYP induction in the presence or absence of Dap treatment. The mice treated with CYP+vehicle exhibited decreased levels of DCs in the spleens and ILNs relative to control-treated mice, while those treated with CYP+Dap exhibited a significant increase in DC numbers in both organs relative to CYP alone (**Figures 4A–C**). In contrast, CYP+vehicle treatment-induced DCs in the UBs, while CYP+Dap treatment reduced the numbers of UBs DCs (**Figures 4A, D**). Thus, Dap differentially mediates DCs in systemic and mucosal organs during IC. The substantial decrease in the number of UB DCs after Dap treatment could reduce T cell activation and might play a role in suppressing the severity of IC.

### Dap Treatment Reduces the Number of Neutrophils During IC

**Figure 5:**
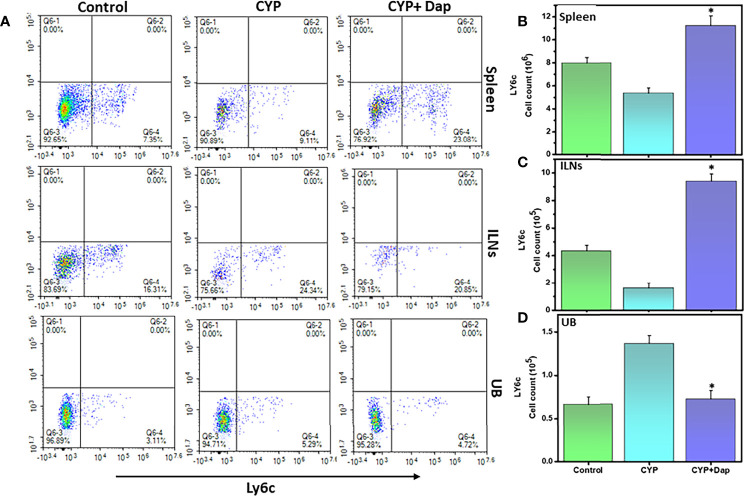
Change in neutrophils frequency after Dap treatment during IC. Single-cell suspensions from the spleen, ILN, and UB were isolated from the three groups at the experimental endpoint, as described in the legend in **Figure 2**. Mean values ± SEM from three independent experiments involving six mice per group (n = 18) are shown. **(A)** Neutrophils were stained for Ly6C expression and analyzed by flow cytometry. Density plots are shown with the mean percentage changes (lower right quadrant) and total number per mouse of the Ly6G cells in the spleen **(B)**, ILN **(C)**, and UBs **(D)**. The statistical significance between the three groups was assessed and asterisks indicate statistically significant differences between CYP+vehicle and CYP+Dap treatment groups (*p < 0.05).

It has been shown that the concentration of neutrophil elastase in the urine is increased and it correlates with pain and bladder function in cystitis patients (33). We, therefore, examined the effects of Dap treatment on the frequency and number of neutrophils in CYP-induced IC in mice. We observed a decrease in the number of neutrophils in the spleens and ILNs and an increase in the UBs after the induction of IC, as compared to control mice (**Figures 5A–D**). However, the number and frequency of neutrophils from spleens and ILNs were significantly increased after CYP+Dap treatment (**Figures 5B, C**). In contrast, the number and frequency of neutrophils decreased after CYP+Dap treatment as compared to CYP alone in UBs (**Figure 5D**). The substantial decrease in the number of neutrophils in UBs by Dap treatment might suppress the severity of CYP-induced IC.

### Dap Reduces the Frequency of Mast Cells (FcεRI+CD117) During CYP-Induced IC

**Figure 6:**
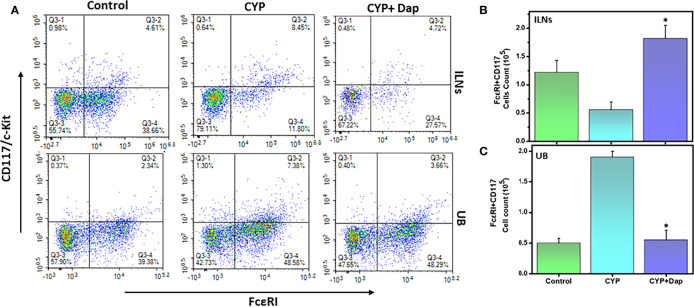
Change in mast cells after Dap treatment during IC. Single-cell suspensions from ILN and UB were isolated from the three groups at the experimental endpoint, as described in the legend in **Figure 2**. Mean values ± SEM from three independent experiments involving six mice per group (n=18) are shown. At the experimental endpoint, mast cells (MCs) were for expression of FcεRI + CD117/c-kit, and analyzed by flow cytometry. Density plots are shown with the mean percentage changes in MC levels **(A)** and the total number of MCs in the ILNs **(B)** and UBs **(C)** per mouse. The statistical significance of flow cytometry data between the three groups was assessed and asterisks indicate statistically significant differences between CYP+vehicle and CYP+Dap treatment groups (^*^p<0.05).

We next examined the changes in the frequency and number of mast cells (MCs) in ILNs and the UBs during CYP-induced IC in the presence and absence of Dap treatment. The number and frequency of MCs (FcεRI+CD117) were decreased in ILNs and significantly increased in the UBs (p<0.01) of CYP+vehicle-treated mice relative to control mice (**Figures 6A–C**) This alteration was significantly reversed in the UBs after CYP+Dap treatment (**Figure 6C**). These data suggest that while the huge number of MCs attracted to the UBs during CYP-induced may be responsible for the progression of disease severity, their suppression by Dap treatment may be responsible for suppressing IC severity and symptoms.

### CYP Differentially Induces Cytokine and Chemokine Responses During IC

**Figure 7:**
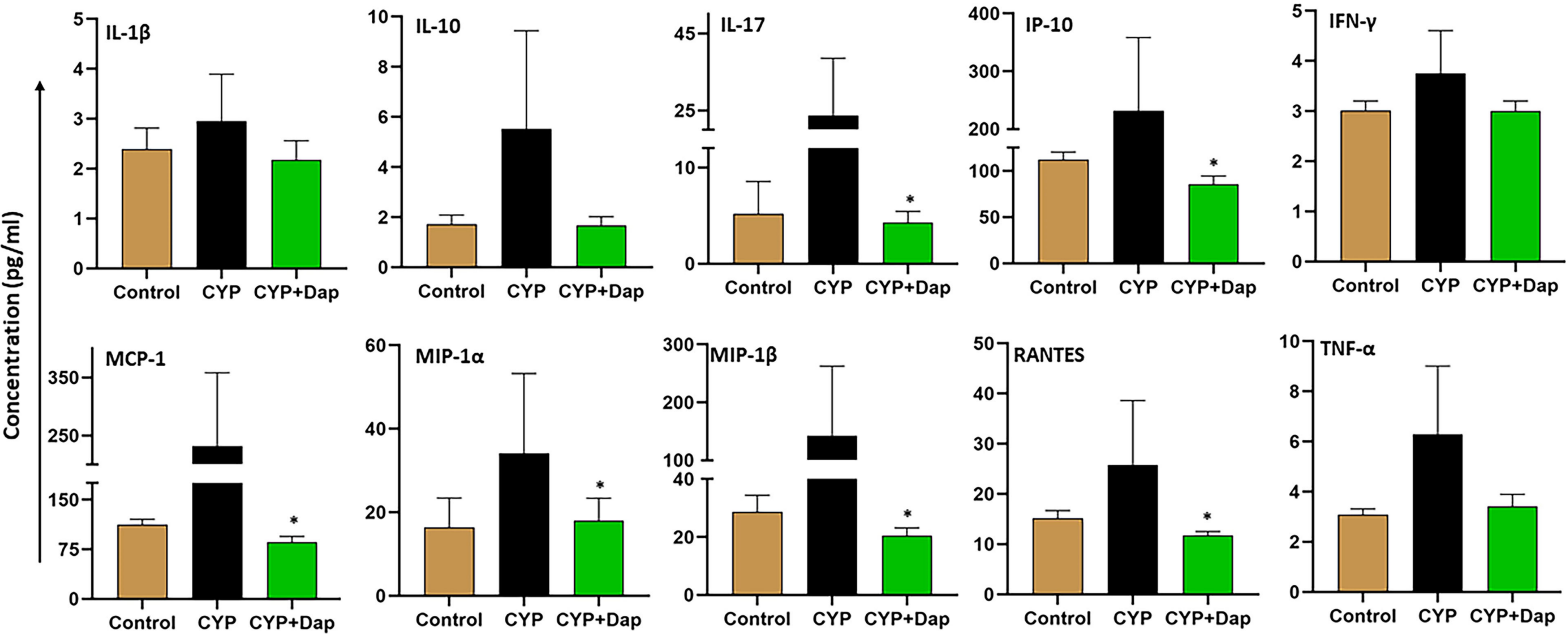
Dap reduces levels of systemic pro-inflammatory cytokines and chemokines in CYP-induced IC. Whole blood was collected from mice in the three groups at the experimental endpoint and sera were produced by centrifugation. Sera were analyzed by ELISA assay for CYP-induced inflammatory cytokines and chemokines IL-1β, MIP-1α, IFN-γ, RANTES, IL-17, MCP-1, IP-10, MIP-1β, IL-10, and TNF-α. Treatment of CYP+vehicle induced systemic inflammatory cytokines, while treatment with CYP+Dap suppressed their expression. Data represent the concentration of inflammatory cytokine and chemokines ± SEM from the three independent experiments. Asterisks indicate statistically significant differences between CYP+vehicle and CYP+Dap treatment groups (*p < 0.05).

Urinary chemokines and cytokines are considered to be the noninvasive predictor of IC (34). We used a Bio-Plex ELISA assay to measure the levels of these chemokines and cytokines in the serum from the three groups of mice described above. CYP-induced mice exhibited elevated levels of systemic C-X-C motif chemokine ligand 10 (CXCL10)/interferon-gamma-inducible protein 10 (IP-10), IL-1β, IL-10, IL-17, interferon-gamma (IFN-γ), monocyte chemoattractant protein-1 (MCP-1), macrophage inflammatory protein-1 alpha (MIP-1α), MIP-1β, RANTES (regulated upon activation, normal T cell expressed and presumably secreted), and TNF-α inflammatory cytokines and chemokines, relative to those in control mice (**Figure 7**). However, CYP+Dap treatment reduced these cytokine and chemokine levels during IC as compared to CYP alone. These data indicate that these pro-inflammatory cytokines play a role in inducing IC, mainly by increasing the different cell-mediated immune responses, while the reduction of these levels by Dap treatment might be responsible at least in part for reduced IC inflammation in the UB.

### Dap Suppresses Various Signaling Pathways to Ameliorate IC

**Figure 8:**
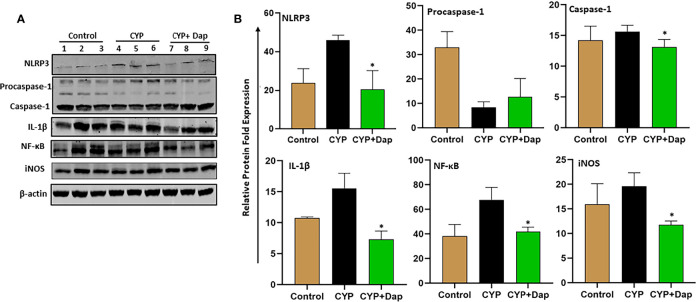
Effect of Dap on NF-kB, NLRP3, iNOS, caspase-1, IL-1b expression during IC. Single UBs cell suspensions were obtained from the three groups at the experimental endpoint, as described in the legend in **Figure 1**. Mean values ± SEM from three independent experiments involving six mice per group (n=18) are shown. **(A)** Cells were lysed, their protein concentrations were determined, and proteins were subjected to immunoblot analysis using antibodies specific for NLRP3, NFkB, iNOS, caspase-1, and IL-1b. **(B)** The statistical significance of immunoblot data between the control (1-3), CYP+ vehicle (4-6), and CYP+ Dap (7-9) groups was assessed as a change in expression of protein as compared to control (normalized with actin). Asterisks indicate statistically significant differences between CYP+ vehicle and CYP+ Dap treated groups (*p < 0.05).

The NLRP3 inflammasome mediates activation of caspase-1 and secretion of IL-1β in response to infection and cellular damage. While nitric oxide suppresses NLRP3 activation and protects against LPS-induced septic shock and reduces cytokine production (35). We, therefore, determined the effect of Dap on the IL-1β and NF-κB signaling pathways in the UBs of each group of mice during IC by immunoblot analysis. Mice that received CYP+vehicle induced the expression of caspase-1, IL-1β, NLRP3, NF-κB, and iNOS, relative to control mice (**Figures 8A, B**). In contrast, CYP+Dap treatment resulted in decreased levels of caspase-1, IL-1β, NLRP3, NF-κB, and iNOS, relative to CYP+vehicle treatment. Taken together, these data suggest that Dap reduces the caspase-1 and NF-κB signaling pathways, thus suppressing the production of cytokines and chemokines, which in turn ultimately reduce the severity of IC.

## Discussion

Despite numerous studies to date, the nature and etiology of IC are still not fully understood. Several efforts focused on the mechanism responsible for IC but were not successful due to the lack of a suitable animal model that mimics human IC. More recently, several autoimmune etiologies have been linked to IC conditions and inflammation has emerged as a key constitutive element in the events cascade leading to IC. For example, the levels of proinflammatory cytokines increase in IC patients (36, 37). We previously showed that inflammatory infiltrates, mainly activated T cells, mast cells, and neutrophils were increased in the UB of mice with CYP-induced IC (32). The NLRP3 inflammasome linked with many inflammatory diseases is expressed by human bladder epithelium cells (38). Thus, a better understanding of the intricate balance between inflammatory disease and expression of the NLRP3 inflammasome, which alters the numbers of neutrophils, mast cells, T cells, DCs, and their signaling pathways, will be required to effectively treat IC. Our goal was to determine whether inhibition of the NLRP3 inflammasome by its selective inhibitor, Dap, suppresses CYP-induced IC. In this study, we demonstrated that inhibiting NLRP3 using Dap reduced the IC disease score, level of systemic cytokines, infiltration of T cells, mast cells, neutrophils, and mediating DCs in the UB, thereby ameliorating the severity of IC.

Many reports have linked IC with several autoimmune diseases (39). We and others have shown that in IC patients and two experimental mouse models, T cells, neutrophils, and mast cells are infiltrated in the UB (16, 32, 40). T cell phenotypes, particularly Th1, are increased during IC (41), and CXCR3-expressing T cells mediate Th1 and inflammatory lymphocytes (42). IC also induces a high number of infiltrating T cells (CD4^+^ and CD8^+^) in the UB (43). In this study, we observed a differential change in the number and frequency of CD4^+^ and CD8^+^ T cells in the spleens and ILNs of mice treated with Dap+CYP, relative to CYP+vehicle. However, numbers of these cells were enhanced in the UBs of CYP-treated mice, as compared to control. Conversely, the NLRP3 inhibitor Dap suppressed the frequency and number of both CD4^+^ and CD8^+^T cells in the UB, suggesting a possible pathway to suppress or remove these infiltrated cells from the UB.

Next, we determined the phenotypes of infiltrated T cells in the UB. We observed that numbers of CXCR3-expressing T cells that increase in CYP-induced IC were significantly reduced (p<0.01) in the spleens and the UBs after Dap treatment. It has been well established that CYP administration leads to immunosuppression, such as killing immune cells, interfering with the proliferation and differentiation of B and T cells, and restraining the humoral and cellular immune response (44). We and others have shown that T cells infiltrated the mucosa of the urinary bladder (UB) of CYP-induced mice (32, 43). Increased CXCR3 expression on activated T cells has been reported previously during inflammatory bowel disease (IBD), relative to controls (16, 45, 46). Similarities between IBD and IC in T-cell and monocyte infiltration (47, 48) suggest a possible pathway that Dap might use to suppress IC symptoms. These leukocyte infiltrations in the bladder during CYP-induced cystitis happen due to highly reactive aldehyde CYP-derived metabolite acrolein mediated loss of bladder urothelium and associated immune response. In this study, we believe that acrolein insults result in epithelial cell damage and enhance infiltration of T cells in the bladder that induce chemokines and cytokines to enhance inflammation. Taken together these suggest that after CYP induction loss of intestinal barrier function alters mucosal immunity and participates in modulating the immune response to enhance IC symptoms in the UB. While Dap reduces the T cells in the UB, responsible in part for suppressing inflammation during IC.

Neutrophils are important effector cells in the innate immune system that are increased in the urine of IC patients (49). Neutrophils react to multiple signals including cytokines and other inflammatory factors that regulate inflammation. The concentration of neutrophil elastase enzyme is elevated in IC patients (33), suggesting a possible relationship with IC severity. Interestingly, NLRP3 regulates neutrophil functions and contributes to hepatic ischemia-reperfusion injury (50). In this study, we observed that the number and percentage of neutrophils were increased in the spleens and ILNs but significantly declined in the UB of mice treated with CYP+Dap, compared to those treated with CYP+vehicle. Our findings suggest a differential function of neutrophils after Dap treatment in systemic and effector sites (UB) by regulating the suppression of other leukocyte functions like mast cells and activated T cells in UB sites for ameliorating IC symptoms. These effects may also be indirectly supported by a reduction in T cells, mast cells, and cytokines levels after Dap treatment.

Mast cells (MCs) are multifunctional innate immune cells that are involved in both clinical and experimental models of IC (51). The number of MCs increases in the UB during IC (52) and produces cytokines and chemokines that mediate neutrophils (10). The number and frequency of MCs gated on FcεRI+CD117 cell surface markers increased in IC patients relative to healthy donors (53). MCs are closely involved with IC severity in UB (51). Interestingly, a recent study showed that activation of MCs by NLRP3 contributed to the development of endometriosis (54). Further, high levels of infiltration by MCs occur in response to the damage to bladder integrity due to frequent urinary toxicity in IC (55). In our study, we observed a significant increase in the number and frequency of MCs in the UB after CYP induction that was suppressed by Dap treatment. In contrast, Dap treatment was associated with increased numbers of MCs in ILNs, suggesting a differential regulation in systemic and mucosal sites that could alter IC severity. Taken together, our data suggest a condition in which CYP induces MCs in the UB sites leading to the development of severe IC. However, Dap treatment reduced the MCs numbers in the UB which may, in turn, lead to a decline in IC severity and symptoms.

Dendritic cells (DCs) are abundant in systemic and mucosal tissue, where they help to regulate the immune response. Activation of DCs is a critical step for antigen presentation that is required for further activation and clonal expansion of T cells (56). On activation of DCs, several chemokines participate in their recruitment to sites of inflammation (57). In this study, we observed that both the frequency and number of DCs increased in the spleens and ILNs and decreased in the UB after CYP+Dap treatment, relative to CYP+vehicle. Our data suggest that Dap may induce DCs to support T cells for clonal expansion and function in the spleens and ILNs while reducing DCs in the UB to suppress T cell functions. However, this data did not allow us to reach any firm conclusions about Dap’s differential function on DCs at the systemic and mucosal sites for mediating T cell function.

Our previous study showed that the levels of several cytokines and chemokines increased in an autoimmune-induced model of cystitis (32) and we have detected increases in systemic levels of TNF-α in other inflammatory models (46). Here, we report decreased levels of TNF-α, IL-17, IP-10, IL-1B, RANTES, MCP-1, IL-10, and IFN-γ in the serum of mice treated with Dap+CYP relative to those in CYP+vehicle-treated mice. Several previous reports suggested that inhibition of the NLRP3 inflammasome attenuates cystic fibrosis, colonic inflammation, and other inflammatory disorders (58–60). Taken together, our data suggest that the involvement of these cytokines and chemokines in the decreased inflammatory response after CYP+Dap treatment might be responsible in part for protection from IC severity.

IL-1β is a clinically-relevant urinary marker for interstitial cystitis (61) and has been identified as an immunotherapeutic target for cystitis (62). NLRP3 inflammasomes are important central mediators of CYP-induced bladder inflammation, and caspase-1 expression is increased in bladder urothelial cells (63, 64). Inflammasome-dependent caspase-1 activation also paves the way for the secretion of several pro-inflammatory cytokines, including IL-1β (65). NLRP3 activation leads to cleavage of procaspase-1 to activated caspase-1, which processes pro-IL-1β to mature, biologically active IL-1β (66). It has been shown that extracellular vesicles derived from mesenchymal stem cells suppress neuroinflammation and mechanical allodynia in rat IC by inhibiting NLRP3 inflammasome activation (67). The suppression of NF-κB pathways by the voltage-gated calcium channel subunit α_2_δ-1 reduces cystitis symptoms and bladder hypersensitivity (68) iNOS plays a role in CYP-induced cystitis in rats (69). Towards, an herbal medicine-derived Shionone alleviates interstitial cystitis in rat model *via* NF-κB, NLRP3 and GSDMD-N mediated pathways (70).

We observed reduced accumulation of IL-1β, caspase-1, NF-κB, and iNOS in the UB and increased accumulation of procaspase-1 in CYP+Dap treated mice, relative to that in mice treated with CYP+vehicle. These data corroborate previous findings and allow us to suggest that the mechanism by which IC severity is suppressed by CYP+Dap treatment may occur through reduction of the systemic and UB levels of caspase-1 and NF-κB pathways and their downstream products IL-1β and iNOS levels. However, we will need further in-depth study by overexpression or knockout of these pathways to draw any prudent conclusions.

In summary, the results of our present study suggest that CYP induction enhances activated T cells, neutrophils, and mast cells that induced increased levels of pro-inflammatory cytokines and chemokines, thereby increasing the severity of IC symptoms. Treatment of mice with Dap reduced these overall cellular immune responses, and levels of cytokines and chemokines including IL-1β, along with caspase-1, NF-κB, and iNOS in the UB, and differentially mediated DCs that regulate the UB response and suppress IC severity. Our results highlight the importance of NLRP3 inhibitors in suppressing IC and present future opportunities for more detailed clinical studies.

## Data Availability Statement

The raw data supporting the conclusions of this article will be made available by the authors, without undue reservation.

## Ethics Statement

The animal study was reviewed and approved by The University of Tennessee health Science Center.

## Author Contributions

US conceived the ideas, edited the manuscript, and performed data analysis. SK performed the work, wrote the manuscript, performed data analysis, and created the figures. RA performed the western blot analysis, created the figures, and edited the manuscript. All authors contributed to the article and approved the submitted version.

## Conflict of Interest

The authors declare that the research was conducted in the absence of any commercial or financial relationships that could be construed as a potential conflict of interest.

## Publisher’s Note

All claims expressed in this article are solely those of the authors and do not necessarily represent those of their affiliated organizations, or those of the publisher, the editors and the reviewers. Any product that may be evaluated in this article, or claim that may be made by its manufacturer, is not guaranteed or endorsed by the publisher.
